# Acute measures of upper thermal and hypoxia tolerance are not reliable predictors of mortality following environmental challenges in rainbow trout (*Oncorhynchus mykiss)*

**DOI:** 10.1093/conphys/coab095

**Published:** 2021-12-23

**Authors:** Nicholas Strowbridge, Sara L Northrup, Madison L Earhart, Tessa S Blanchard, Patricia M Schulte

**Affiliations:** 1 Department of Zoology University of British Columbia, Vancouver, BC V6T 1 Z4, Canada; 2 Freshwater Fisheries Society of British Columbia, Abbotsford, BC V9A 7S2, Canada

**Keywords:** upper thermal tolerance, inter-individual variation, ILOS, hypoxia tolerance, fish, CTmax, Climate change

## Abstract

Anthropogenic climate change threatens freshwater biodiversity and poses a challenge for fisheries management, as fish will increasingly be exposed to episodes of high temperature and low oxygen (hypoxia). Here, we examine the extent of variation in tolerance of acute exposure to these stressors within and among five strains of rainbow trout (*Oncorhynchus mykiss*) currently being used or under consideration for use in stocking programmes in British Columbia, Canada. We used incipient lethal oxygen saturation (ILOS) as an index of acute hypoxia tolerance, critical thermal maximum (CT_max_) as an index of acute upper thermal tolerance and mortality following these two acute exposure trials to assess the relative resilience of individuals and strains to climate change-relevant stressors. We measured tolerance across two brood years and two life stages (fry and yearling), using a highly replicated design with hundreds of individuals per strain and life stage. There was substantial within-strain variation in CT_max_ and ILOS, but differences among strains, although statistically significant, were small. In contrast, there were large differences in post-trial mortality among strains, ranging from less than 2% mortality in the most resilient strain to 55% mortality in the least resilient. There was a statistically significant, but weak, correlation between CT_max_ and ILOS at both life stages for some strains, with thermally tolerant individuals tending to be hypoxia tolerant. These data indicate that alternative metrics of tolerance may result in different conclusions regarding resilience to climate change stressors, which has important implications for stocking and management decisions for fish conservation in a changing climate.

## Introduction

Global climate change and other human-induced habitat alterations are causing drastic declines in freshwater biodiversity (Comte & Olden, 2017; Ficke, Myrick, & Hansen, 2007; Jenkins, 2003; Reid *et al.*, 2019), with increasing temperatures being particularly important for cold-water fish (Comte, Buisson, Daufresne, & Grenouillet, 2013; Eaton & Scheller, 1996; Wenger *et al.*, 2011). Increased temperatures are associated with declines in dissolved oxygen (DO; hypoxia) because high temperatures have the dual effect of decreasing oxygen solubility and increasing oxygen consumption by microorganisms (Diaz & Breitburg, 2009). High nutrient loading from agricultural activities exacerbates these effects by increasing microbial metabolism, which causes further decreases in DO levels. The resulting hypoxic episodes can be devastating for fish at a time when their own oxygen demand is elevated due to high temperatures (Diaz & Breitburg, 2009). This combined effect of hypoxia and high temperatures, which is becoming increasingly frequent in aquatic ecosystems as a result of human activities (Diaz & Breitburg, 2009; Jenny *et al.*, 2016; O’Reilly *et al.*, 2015), is likely to be a primary driver of changes in habitat suitability for fishes (Deutsch, Ferrel, Seibel, Pörtner, & Huey, 2015). These changes will thus present a substantial challenge for the conservation and management of freshwater fish into the future.

Organisms have only a few fundamental ways in which to cope with the increasing temperatures and episodes of hypoxia in freshwater. They can move to less stressful habitats, exhibit phenotypic plasticity or adapt *in situ* (Gienapp, Teplitsky, Alho, Mills, & Merilä, 2008)*.* Phenotypic plasticity may be limited in its effectiveness in response to climate stressors (DeWitt, Sih, & Wilson, 1998; Seebacher, White, & Franklin, 2015), and we will not consider it further here. Similarly, migration is not an option for many freshwater organisms either because of the natural structure of the habitat or due to habitat fragmentation from damming (Strayer & Dudgeon, 2010). On the other hand, human-assisted migration, which involves passively or actively moving species or strains outside their currently occupied ranges, has been proposed as a means of addressing the impacts of climate change (Hewitt *et al.*, 2011; Vitt, Havens, & Hoegh-Guldberg, 2009). This approach is not without risk and will require a deep understanding of the relative resilience of different species or strains to climate change stressors. Finally, although evolutionary adaptation has often been considered to be too slow to be relevant in the context of anthropogenic warming, more recent data suggest that rapid adaptation is possible (Munday, Donelson, & Domingos, 2017) and will likely play a key role in the survival of freshwater organisms into the future (Somero, 2010). Rapid adaptation typically occurs from standing genetic and phenotypic variation within strains (Barrett & Schluter, 2008), but for many species, we have little knowledge of the extent of within-species genotypic and phenotypic variation for traits that are relevant to climate change resilience. Without this information, it is difficult to determine whether adaptation is likely to occur for any given trait. Thus, in the context of both estimating the likelihood of adaptation *in situ* and evaluating the need for, and likely success of, assisted migration, characterizing both within and between strain variations in traits related to an organism’s response to climate change will be vital for conservation and management programmes going forward. Indeed, it is becoming increasingly clear that analyzing strain-specific responses to climate-change relevant stressors will be critical in developing appropriate mitigation and conservation efforts to protect species as our climate warms (e.g. Chen *et al.*, 2015; Eliason *et al.*, 2011; Roze, Christen, Amerand, & Claireaux, 2013; Verhille, English, Cocherell, Farrell, & Fangue, 2016; Zhang, Healy, Vandersteen, Schulte, & Farrell, 2018).

Here, we examine within- and among-strain variation in thermal and hypoxia tolerance in rainbow trout (*Oncorhynchus mykiss*), a fish species that is widely stocked for recreational purposes globally and is now present on six out of the seven continents (MacCrimmon, 1972; Stanković, Crivelli, & Snoj, 2015). Rainbow trout are native to lakes, rivers and streams on the western coast of North America. In British Columbia, which encompasses most of the native range of rainbow trout in Canada, this species is widely stocked to support the recreational fishing industry. This industry is of substantial socio-economic importance, contributing ~$8 billion annually to the Canadian economy with close to $1 billion in British Columbia alone (Bailey & Sumaila, 2012; GSGislason & Associates Ltd, 2009; Northrup, 2017). In British Columbia, the Freshwater Fisheries Society of British Columbia (FFSBC), stocks ~ 800 lakes province-wide with ~ 8 million fish, of which 5 million are rainbow trout (Bailey & Sumaila, 2012; Northrup, 2017). Such stocking programmes help to preserve recreational fisheries and promote the survival of natural populations through the alleviation of fishing pressure on wild stocks (Cooke & Cowx, 2006; Cowx, 1994; Froehlich, Gentry, & Halpern, 2017).

Most studies in conservation physiology use relatively modest sample sizes, which provide limited information on both within and between strain variations. Similarly, many studies are performed using wild-caught individuals, which makes it difficult to distinguish genetic differences from variation due to various forms of plasticity and epigenetic effects. This distinction is critical for determining the potential for evolutionary adaptation (Crozier & Hutchings, 2014; Gienapp *et al.*, 2008; Merilä & Hendry, 2014). In contrast, in this study we reared five different strains of rainbow trout in common conditions from fertilization, thus minimizing the potential effects of phenotypic plasticity, used 8–25 families per strain to capture a significant fraction of within-strain genetic variation and used large numbers of individuals of each strain (~100 to ~ 500) to characterize the range of phenotypic variation among individuals within a strain.

We used critical thermal methodology to assess upper thermal tolerance (measured as CT_max_). CT_max_ is a dynamic measure of acute upper thermal tolerance in which temperature is increased over a relatively short period of time until an organism exhibits loss of equilibrium (LOE; inability to maintain dorso-ventral orientation) and is unable to escape from conditions that would otherwise lead to death (Becker & Genoway, 1979; Cowles & Bogert, 1944; Lutterschmidt & Hutchison, 1997).While CT_max_ is a measure of acute and not chronic thermal tolerance (McKenzie *et al.*, 2020), we chose CT_max_ for our assessment of thermal tolerance because it is considered to be a non-lethal measure, it has been shown to be highly repeatable at the individual level (Grinder, Bassar, & Auer, 2020; Morgan, Finnøen, & Jutfelt, 2018; O’Donnell, Regish, McCormick, & Letcher, 2020) and it can easily be assessed on a large number of individuals. Although the direct ecological relevance of CT_max_ has been debated_,_ there is some evidence that it correlates to a more ecologically relevant tolerance to slower warming rates and it is a predictor of the global distribution of fish species (Åsheim, Andreassen, Morgan, & Jutfelt, 2020; Sunday, Bates, & Dulvy, 2011).

To provide an index of acute hypoxia tolerance, we used a hypoxia challenge test (HCT), which measures the incipient lethal oxygen saturation (ILOS) or the percent DO saturation at which an organism exhibits LOE (Roze *et al.*, 2013). We chose ILOS because, like CT_max_, it provides a non-lethal measure of tolerance, it is highly repeatable (Nelson, Kraskura, & Lipkey, 2019; Rees & Matute, 2018) and it can easily be applied to large numbers of individuals. We also assessed mortality after the fish were exposed to a hypoxia tolerance test followed by a thermal tolerance test several weeks later as an alternative index of climate-change resilience. Although both CT_max_ and ILOS are considered to be non-lethal methods (Cowles and Bogert, 1944; Becker & Genoway, 1979; Claireaux *et al.*, 2013), few studies have examined the effects of determining both types of tolerance in the same individual, and thus the extent of mortality in repeated trials is not known. In natural environments fish may experience repeated exposure to these stressors, and thus data on the level of resilience to this type of more complex stressor exposure are urgently needed.

In this study, we address the following questions: (i) How much within-strain variation is present in CT_max_ and ILOS? (ii) Is there a difference in CT_max_ and ILOS among the strains? (iii) Are differences among strains in CT_max_ and ILOS reflected in differences in mortality following stressor exposure? (iv) Is there a correlation between CT_max_ and ILOS at the level of individuals? The conservation goal of this work is to aid in the identification of strains that may be resilient to high temperatures and low environmental oxygen. More broadly, this work also allows identification of the extent of inter-individual variation in multiple traits that are relevant to climate change resilience, and which may provide a substrate for adaptation in the face of climate change.

## Methods

### Experimental animals

We examined variation in upper thermal tolerance and hypoxia tolerance in five strains of rainbow trout currently being used or under consideration for use in stocking programmes across two brood years (2017 and 2018) and two life stages (fry and yearling). Strains assessed include the wild-derived Blackwater River (BW) strain, a riverine piscivorous strain (Scott, Dhillon, Schulte, Richards, & Magnan, 2015) that is now reared by the FFSBC in a broodstock lake (FFSBC, 2021); the Carp Lake (CL) strain, a highly competitive lake-dwelling strain (FFSBC, 2021); the Pennask Lake (PN) strain, an insectivorous, non-competitive lake-dwelling strain (FFSBC, 2021); the Horsefly River (HF) strain, a large, late-maturing Quesnel Lake strain that utilizes the Horsefly River for spawning and rearing opportunities (Holmes, 2009); and the Fraser Valley (FV) strain, a domesticated strain that has been used in the British Columbian recreational fish stocking programme since the 1960s (FFSBC, 2021). Not all strains were used for each brood year and/or life-stage (see Tables 1 and 2).

**Table 1:** Trial dates, sample sizes and trial numbers for all strains in the 2017 brood year (Experiments 1 and 2)

Strain	Trial dates (fry)	Sample size (fry) Hypoxia thermal		Trial dates (yearling)	Sample size (yearling) Hypoxia thermal	
Blackwater River (BW)	**Thermal:** Nov./Dec. 2017 **Hypoxia:** Feb. 2018	1 trial 100 fish/trial *n* = 100	2 trials 50 fish/trial *n* = 100	Apr./May 2018	5 trials 99–101 fish/trial *n* = 500	5 trials 99–100 fish/trial *n* = 500
Carp Lake (CL)	**Thermal:** Nov./Dec. 2017 **Hypoxia:** Feb. 2018	1 trial 99 fish/trial *n* = 99	2 trials 50 fish/trial *n* = 100	Apr./May 2018	4 trials 100 fish/trial *n* = 400	4 trials 100 fish/trial *n* = 400
Fraser Valley (FV)	June 2018	1 trial 99 fish/trial *n* = 99	1 trial 99 fish/trial *n* = 99	Aug. 2018	5 trials 99–100 fish/trial *n* = 500	5 trials 99–100 fish/trial *n* = 500
Pennask Lake (PN)	N/A	N/A	N/A	June 2018	1 trial 100 fish/trial *n* = 100	1 trial 97 fish/trial *n* = 97

**Table 2:** Trial dates, sample sizes and trial numbers for all strains in the 2018 brood year (Experiment 3)

Strain	Trial dates (fry)	Sample size (fry) Hypoxia thermal		Trial dates (yearling)	Sample size (yearling) Hypoxia thermal	
Blackwater River (BW)	Oct. to Dec. 2018	14 trials 28–38 fish/trial *n* = 489	14 trials 25–37 fish/trial *n* = 436	Apr./May 2019	3 trials 33–34 fish/trial *n* = 100	3 trials 33–34 fish/trial *n* = 100
Carp Lake (CL)	Oct. to Dec. 2018	14 trials 29–34 fish/trial *n* = 487	14 trials 28–34 fish/trial *n* = 425	Apr./May 2019	3 trials 33–34 fish/trial *n* = 100	3 trials 33–34 fish/trial *n* = 100
Horsefly River (HF)	Oct. to Dec. 2018	14 trials 30–36 fish/trial *n* = 497	14 trials 28–36 fish/trial *n* = 453	Apr./May 2019	3 trials 33–34 fish/trial *n* = 100	3 trials 33 fish/trial *n* = 99
Pennask Lake (PN)	Feb. 2019	5 trials 100 fish/trial *n* = 500	5 trials 97–100 fish/trial *n* = 495	N/A	N/A	N/A

Following the acquisition of milt and eggs from adults residing in broodstock lakes (CL and BW; FV adults housed at FFSBC, Duncan, BC hatchery) or wild lakes (HF and PN), we bred 25 independent families (25 females and 25 males) per strain for the respective brood years of BW, CL, PN and FV, and 8 families (8 females and 7 males) for HF because of their small population size and difficulty of capturing wild individuals in Quesnel Lake. These strains spawn at different times of the year and thus crosses for BW, CL and HF were performed in May; for PN in June; and for FV in October of each brood year. Following fertilization, eggs were treated with ovadine, counted out evenly to represent all mothers and then pooled by strain for incubation. Eggs were raised in heath trays in the dark until hatch (~6–8 weeks post-fertilization) with flow-through well water (10–12°C). After hatch, fry from each strain were housed separately in 2400 L tanks containing 10–12°C flow-through well water and kept under a natural photoperiod of Abbotsford, BC. From yolk-sac absorption to ~ 5 g, the fish were fed to satiation multiple times a day with Bio-Oregon BioVita #0 to #2 and with Bio-Oregon Bio-Clark’s fry 1.2 mm thereafter (Bio-Oregon 2020). All offspring were maintained as diploids (2n) and housed and experimented on at the Fraser Valley Trout Hatchery (Abbotsford, BC).

Some experiments were performed using a repeated measures design in which the same individuals were tested for both upper thermal and hypoxia tolerance (see Experimental design and statistical analyses section). For these repeated measures trials, fish were individually tagged with Biomark GPT12 (12 mm) passive integrated transponder (PIT) tags. Briefly, individual fish were anesthetized with 50–100 mg/L (depending on size) of MS-222 (Tricaine methanesulfonate; buffered 1:1.5 with NaHCO_3_), length and weight were measured, then a small incision was made along the bottom of the fish slightly anterior to the anal fins and a PIT tag was inserted, read for identification and the fish was returned to their holding tank (500 L).

All experiments were performed according to approved University of British Columbia animal use protocol A16-0329.

### Hypoxia tolerance (measured as ILOS)

We measured ILOS using an HCT as outlined by Claireaux *et al.*, (2013). Note that ILOS is inversely related to hypoxia tolerance, with high ILOS indicating poor hypoxia tolerance. Fish were transferred from holding tanks to the assessment tank (215 L) and left for 30 min to adjust to the testing apparatus before the beginning of each trial. All hypoxia trials started between 9 and 11 am and were conducted at the fish’s holding temperature of 11°C ± 1°C. During each trial, the DO was lowered by bubbling nitrogen into the experimental tank such that DO decreased by ~ 1.5% DO saturation min^−1^ until ~ 20% DO saturation was reached. After ~ 20% DO saturation was reached, the rate of decrease was lowered to 0.1% DO saturation min^−1^ until the end of the trial. A small circulation pump was placed in the experimental tank to ensure consistent mixing of the added nitrogen. Hypoxia tolerance was determined as the DO saturation (% sat.) at which an individual fish experienced LOE. Once LOE was reached the fish was removed from the experimental tank, scanned for PIT tag identification where applicable and placed into a recovery tank containing fresh, fully aerated water at their holding temperature. Following measurement of hypoxia tolerance, the fish involved in non-repeated measures trials were immediately sacrificed and fin-clipped, whereas fish involved in repeated measures trials were allowed to recover for either 2 or 3 weeks (depending on the experiment; see Experimental design and statistical analyses\ section) before determination of upper thermal tolerance.

### **Upper thermal tolerance (measured as CT**_**max**_ **)**

We assessed upper thermal tolerance using a critical thermal maximum (CT_max_) protocol modified from Beitinger, Bennet, and McCauly (2000). The day prior to the experiment, a 500-L tank adjacent to the assessment tank was heated to ~ 40–45°C for use as a source of heated water. All trials started between 9 and 11 am. At the start of each trial, fish were transferred from their holding tanks to the assessment tank (250 L) and left undisturbed for 30 min to adjust to the testing apparatus. A small circulation pump was placed in the assessment tank to avoid thermal stratification of the water and to achieve consistent mixing of added water. Beginning at 11°C ± 0.5°C, water temperature was increased by pumping water from the hot-water tank into the assessment tank through a polyvinyl chloride (PVC) pipe fitted with a flow valve. Throughout each trial, small adjustments were made to the flow to achieve a consistent ramping rate of 0.3°C min^−1^ until the water reached 18°C. At 18°C, the flow was slowed considerably to achieve a ramping rate of 0.1°C min^−1^ for the remainder of the trial. This two-stage approach was chosen to allow maximum discrimination among individuals while allowing the trial to be completed within ~ 3 h. Upper thermal tolerance (CT_max_) was measured as the temperature at LOE. After LOE, each fish was removed from the experimental tank, scanned for PIT tag identification where applicable and placed in a recovery tank containing fresh ~ 21°C water. Over the course of 2 h post-trial, the temperature in the recovery tank was slowly brought down to the acclimation temperature (~11°C). This gradual recovery protocol was adopted because preliminary experiments suggested that post-trial mortality was extremely high if fish were immediately transitioned from their temperature at CT_max_ to their acclimation temperature. Following measurement of upper thermal tolerance, the fish were sacrificed and fin-clipped for non-repeated measures trials, allowed to recover for ~ one week to collect post-trial mortality data or allowed to recover for 3 weeks and had their hypoxia tolerance measured (depending on the experiment, see hereafter).

### Experimental design and statistical analyses

All statistical analyses for all experiments were carried out in R (version 1.1.456; R Core team (2021)). Alpha was set at 0.05 throughout. Prior to analysis, all data were tested for homogeneity of variance and normality and transformed if required. Graphs were generated using ggplot2 and ggpubr (Kassambara, 2020; Wickham, 2016).

**Experiment 1:** This experiment utilized fry of the 2017 brood year from the BW, CL and FV strains (Table 1). Note that the PN strain was not examined at the fry stage because this strain experienced unusually high levels of mortality during rearing and all remaining fish were reared to the yearling stage for use in experiment 2. The primary purpose of experiment 1 was to assess the feasibility of performing trials on large numbers of individuals simultaneously and to examine levels of variation within a strain, thus statistical comparisons of CT_max_ and ILOS among strains were not performed. Fish were not tagged, and different individuals were used for assessment of ILOS and CT_max_. Each strain was tested in a different trial, and either one trial of ~ 100 fish or two trials of ~ 50 fish each were performed per strain (Table 1). The FV strain, which breeds at a different time of year and has a much higher growth rate, was tested at a different time of year (Table 1) to allow testing at a similar body size among strains (Supplementary Fig. S1; Supplementary Table S1)). This means that FV fry were tested under a different photoperiod but similar temperature to the other strains because the hatchery has a constant-temperature water supply (10–12°C year-round), but natural photoperiod. Fry were tested at either 16:8 (FV) or 8:16 (BW or CL).

**Figure 1:**
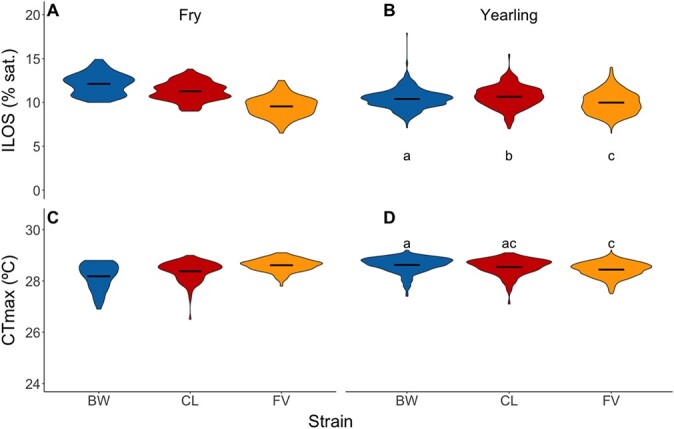
Variation in hypoxia tolerance (ILOS) and upper thermal tolerance (CT_max_) within and among strains in the 2017 brood year (experiments 1 and 2). (**a**) ILOS for fry, (**b**) ILOS for yearlings, (**c**) CT_max_ for fry, (**d**) CT_max_ for yearlings. BW, Blackwater strain (in blue); CL, Carp Lake strain (in red); FV, Fraser Valley Domestic strain (in orange). Black bars indicate mean of each strain. For sample sizes, see Table 1. Differences between strains were not statistically compared for fry because the tolerance of each strain was assessed separately in either one or two trials (Table 1). For yearlings, where multiple trials were performed for each strain, data were analyzed using nested linear mixed effect models followed by Tukey pairwise comparisons (α = 0.05). Significant differences are indicated by dissimilar letters. Data for the PN (yearling) are presented in Supplementary Table S5.

To examine whether the strains differed in the extent of variation in CT_max_ or ILOS, we utilized a Levene’s test for homogeneity of variance using the R package car (Fox & Weisberg, 2019).

**Experiment 2:** At the yearling stage for the 2017 brood year, ILOS, CT_max_ and post-trial mortality were assessed for tagged individuals from the BW, CL, PN and FV strains (Table 1). The primary goal of these experiments was to determine whether hypoxia tolerance and upper thermal tolerance could be assessed on the same individuals and whether independent trials resulted in similar estimates of population trait means. One week prior to the beginning of the trials, all individuals were PIT-tagged and moved to holding tanks (500 L), with each strain held separately. For BW, CL and FV, strains were tested in five separate trials for each strain (although only four trials were completed for CL because of equipment malfunction; Table 1). Only a single trial was performed for PN because this strain experienced unusually high mortality during rearing and fish numbers were limited. ILOS was assessed first, then the fish were allowed 2 weeks of recovery before the determination of CT_max_, followed by assessment of post-trial mortality over the next week. The FV and PN fish were tested at different times of year than the other strains (Table 1) because of the difference in their spawn timing. However, because the BW and CL strains were tested in spring and FV was tested in the summer, all three strains were tested at a photoperiod of 14:10, albeit at different times of year, whereas the PN strain was tested at 16:8 (light:dark). Comparisons among strains tested at different times of year or at different photoperiods should be viewed with caution, as seasonal effects may influence CT_max_ (Lutterschmidt & Hutchison, 1997).

First, the effect of trial group within a strain for CT_max_ and ILOS was analyzed using one-way analysis of variance (ANOVA). Then CT_max_ and ILOS were compared among strains at the yearling stage for the 2017 brood with linear mixed effects models using the lme4 R package (Bates, Mächler, Bolker, & Walker, 2015) with strain as a fixed factor and trial group (nested within strain) as random factor followed by Tukey pairwise comparisons using the lsmeans R package (Lenth, 2016) for all strains except PN, as this strain was tested in only a single trial. Post-trial mortality was computed for each replicate trial within a strain, and the mean mortality was then compared among strains using one-way ANOVA. Correlations between ILOS and CT_max_ were assessed for each strain using Kendall rank correlations.

**Experiment 3:** At the fry and yearling stage for the 2018 brood year, ILOS, CT_max_ and post-trial mortality were assessed for tagged individuals from the BW, CL, PN and HF strains (Table 2). The primary goal of these experiments was to robustly examine whether there was significant variation in upper thermal and hypoxia tolerance among strains. This experiment was conducted in two different groups, with the PN strain assessed separately and the BW, CL and HF strains tested in common garden. One week prior to the beginning of the trials all individuals were PIT-tagged and then held (130-L tanks for fry; 500-L tanks for yearling) until testing. PN fry were assessed in five separate trials with ~ 100 individuals per trial (Table 2). BW, CL and HF fry were assessed across 14 trials, with ~ 33 individuals from each strain in a trial for a total of 400–500 fry per strain, and BW, CL, and HF yearlings were assessed across three trials, with ~ 33 individuals from each strain per trial for a total of ~ 100 yearlings per strain (Table 2). ILOS was determined first, then the fish were allowed three weeks of recovery before the determination of CT_max_, followed by one week of recovery during which post-trial mortality was assessed.

Differences in tolerance among strains were assessed using linear mixed effects models with strain as a fixed factor and trial group as a random factor followed by Tukey pairwise comparisons, as above. Post-trial mortality was computed for each replicate trial within a strain, and the mean mortality was then compared among strains within a brood year and life-stage using one-way ANOVA. ILOS, CT_max_ and post-trial mortality were also assessed for the PN strain (at the fry stage only; ~ 100 fish across five trials, Table 2), but as these assessments were performed at a different time of year due to the difference in its spawning time, we did not make a statistical comparison of PN upper thermal and hypoxia tolerance with the other strains. Correlations between ILOS and CT_max_ were assessed for each strain as described for experiment 2.

**Experiment 4:** At the yearling stage for the 2018 brood year, we assessed tagged individuals to examine the effect of the order of tolerance assessments using additional individuals from the BW and CL strains. We assessed CT_max_ first, followed by 3 weeks of recovery before the determination of ILOS. Fish were assessed in two common garden trials consisting of ~ 50 individuals each, for a total of ~ 100 individuals per strain (Table 3). These data were compared to data for the same strains from experiment 3 (in which ILOS was determined first, followed by CT_max_) using linear mixed effects models with trial order as a fixed factor and trial group as a random factor followed by Tukey pairwise comparisons. Note that all data used for the trial order comparisons were collected within the same week (Table 2 and 3).

**Table 3:** Trial dates, sample sizes and trial numbers for trial order experiment (Experiment 4)

Strain	Trial dates (ILOS then CT_max_)	Sample size (fry) Hypoxia Thermal		Trial dates (CT_max_ then ILOS)	Sample size (yearling) Thermal Hypoxia	
Blackwater River (BW)	Apr./May 2019	3 trials 33–34 fish/trial *n* = 100	3 trials 33–34 fish/trial *n* = 100	Apr./May 2019	2 trials 50 fish/trial *n* = 100	2 trials 43–44 fish/trial *n* = 87
Carp Lake (CL)	Apr./May 2019	3 trials 33–34 fish/trial *n* = 100	3 trials 33–34 fish/trial *n* = 100	Apr./May 2019	2 trials 50 fish/trial *n* = 100	2 trials 49–50 fish/trial *n* = 99

Note: ILOS then CT_max_ trial data are from Experiment 3.

### Results

All raw data are provided in Supplementary data files S1 and S2. The mean wet masses of the fish for each strain and life stage (and tolerance assessment for experiment 1 when these assessments were done on different groups of fish) are provided in Supplementary Figs. S1 and S2 (see online supplementary material for a colour version of this figure) and Supplementary Tables S1 and S2. There was no consistent effect of mass on either CT_max_ or ILOS (Supplementary Tables S3 and S4) and thus fish mass was not considered in subsequent analyses.

### **Within-strain variation in ILOS and CT**_**max**_  **(experiment 1)**

Hypoxia tolerance varied by ~ 5% saturation between the best and worst performing fish within a strain at both life stages for the 2017 brood year (excluding outliers beyond 1.5 of the data interquartile range; Fig. 1a,b). Taking the oxygen ramping rate into account, this translates into a difference of ~50 min before LOE occurs under extreme hypoxia. Thermal tolerance also exhibited substantial variation within strains at both life stages (Fig. 1c,d; Supplementary Table S5), varying by as much as 1.5°C between high and low performers within a strain (excluding outliers beyond 1.5 of the data interquartile range). At the fry life stage, there was no difference in the variance of ILOS among strains (*P* = 0.6178; *F*(2, 295) = [0.482],). However, there were differences in the variance of CT_max_ among strains (*P* = 4.42 × 10^−7^, *F*(2, 296) = [15.379]).

### **Effect of trial on ILOS and CT**_**max**_  **(experiment 2)**

Analysis of the effect of trial on CT_max_ and ILOS in the 2017 brood yearlings revealed small but statistically significant differences in tolerance between trials within some strains (Supplementary Table S6 and S7; ILOS: *F*(4, 495) = [2.476], *P* = 4.35 × 10^−2^ for BW; *F*(3, 393) = [53.88], *P* < 2.00 × 10^−16^ for CL; *F*(4, 494) = [3.074], *P* = 1.61 × 10^−2^ for FV. CT_max_: *F*(4, 494) = [1.524], *P* = 1.94 × 10^−1^ for BW; *F*(3, 396) = [10.5], *P* = 1.17 × 10^−6^ for CL; *F*(4, 494) = [5.142], *P* = 4.57 × 10^−4^ for FV). However, one ILOS trial for CL differed substantially from the rest, which may have been due to inaccurate calibration of the DO meter. Data from this anomalous trial were excluded from subsequent analysis. The fact that statistically significant differences can be detected among trials when sample sizes are large emphasizes the importance of testing the tolerance of all strains within the same trial to accurately determine if there are differences in tolerance among strains.

### **Among-strain variation in CT**_**max**_  **and ILOS (experiments 2 and 3)**

Because we performed multiple trials per strain at the yearling stage for the 2017 brood year (Table 1), we were able to make statistical comparisons among strains, using trial group as the unit of replication. There were significant differences among strains in upper thermal tolerance (Fig. 1D; *P* = 2.07 × 10^−3^; Supplementary Table S5), with the BW strain having a higher CT_max_ than the FV strain. Similarly, there were significant differences among strains in hypoxia tolerance (Fig. 1B; *P* = 2.14 × 10^−4^; Supplementary Table S5), with the FV strain having the greatest tolerance and CL strain having the lowest. Note that these comparisons should be viewed with caution, as the FV strain was tested at a different time of year than the other two strains in an attempt to test the fish at a similar size, although the FV strain was slightly larger at the time of testing (Table 1 and Supplementary Fig. S2).

**Figure 2:**
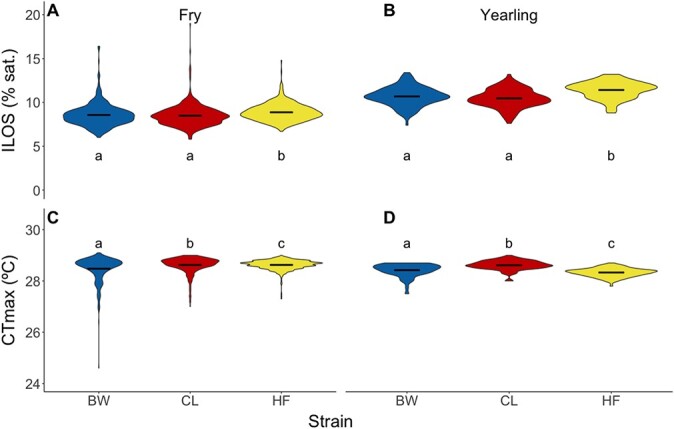
Hypoxia tolerance (ILOS) and upper thermal tolerance (CT_max_) for the 2018 brood year (experiment 3). (**a**) ILOS for fry, (**b**) ILOS for yearlings, (**c**) CT_max_ for fry, (**d**) CT_max_ for yearlings. BW, Blackwater strain (in blue); CL, Carp Lake strain (in red); HF, Horsefly strain (in yellow). Black bars indicate mean of each strain. For sample sizes see Table 2. All data were analyzed using linear mixed effects models with Tukey pairwise comparisons (α = 0.05). Significant differences are indicated by dissimilar letters. Data for the PN (fry) are presented in Supplementary Table 8.

To more robustly examine among-strain variation we used a common garden testing approach in the 2018 brood year (see experiment 3). Note we did not use the FV strain because of the difference in spawn timing and replaced it with HF (Table 2). We also tested the PN strain (at the fry stage only) in separate trials, and these data can be found in Supplementary Table S8. As was the case for the 2017 brood year, we detected substantial variation in both hypoxia tolerance and upper thermal tolerance within strains at both life stages (Fig. 2a–d). We also found small but statistically significant differences among the BW, CL and HF strains in upper thermal tolerance and hypoxia tolerance at both life stages (Fig. 2a–d; fry: *P* = 1.38 × 10^−9^ for ILOS, *P* = 1.22 × 10^−7^ for CT_max_; yearling: *P* = 3.51 × 10^−10^ for ILOS, *P* < 2.20 × 10^−16^ for CT_max_; Supplementary Table S8).

The rank order of hypoxia tolerance among strains was consistent across life stages, with the HF strain having lower hypoxia tolerance (highest ILOS) than the other strains for both fry and yearling (Fig. 2). At the fry stage, mean ILOS differed by 0.4% DO saturation between the most and least tolerant strains and by 0.9% DO saturation at the yearling stage. By contrast, the rank order of upper thermal tolerance (CT_max_) among strains was not consistent across life stages (Fig. 2). Although the CL strain had the highest CT_max_ across both life stages, the HF strain had the lowest CT_max_ at the yearling, but not at the fry life stage. However, it is important to emphasize that differences in mean CT_max_ among strains were extremely small (0.1°C in fry and 0.3°C in yearlings) relative to the large within-strain variation in this trait and likely were only detectable statistically because of the very large sample sizes analyzed here.

Although formal statistical comparisons are not appropriate for the PN strain, in general, this strain was somewhat less tolerant of both hypoxia and high temperature than the other strains across both brood years (Supplementary Table S5 and S8), but any comparisons with this strain should be viewed with caution because these trials were performed separately and at a different time of year.

### Mortality following stressor exposure (experiments 2 and 3)

**Figure 3:**
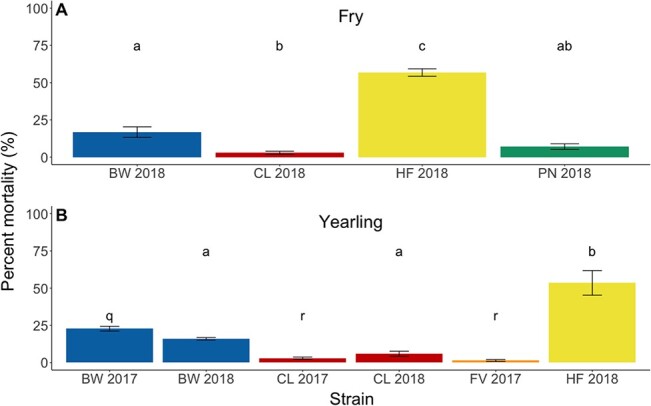
Percent mortality in rainbow trout that had experienced a hypoxia tolerance trial followed by an upper thermal tolerance trial (experiments 2 and 3). (**a**) Percent mortality for fry, (**b**) Percent mortality for yearling. BW, Blackwater strain (in blue); CL, Carp Lake strain (in red); FV, Fraser Valley strain (in orange); HF, Horsefly strain (in yellow); PN, Pennask Lake strain (in green). Values are mean of percent mortality from the replicate trials for each strain/life stage ± SEM. *n* = number of trials, which varied depending on the strain and life stage from 3 to 15 (Tables 1 and 2). Differences in mortality within a brood year were analyzed by one-way ANOVA with Tukey-HSD pairwise comparisons (α = 0.05). Significant differences between strains are indicated by dissimilar letters, with q-r for the 2017 brood year and a–c for the 2018 brood year.

We assessed mortality in the 7 days following the upper thermal tolerance trials in experiments using tagged fish (yearlings in the 2017 brood year and both life stages in the 2018 brood year). In both fry and yearling, there were significant differences in post-trial mortality between strains (Fig. 3; fry: *F*(3, 46) = [90.79], *P* < 2.00 × 10^−16^ for 2018; yearling: *F*(2, 11) = [118.5], *P* = 3.62 × 10^−8^ for 2017; *F*(2, 6) = [26.25], *P* = 1.08 × 10^−3^ for 2018). At both the fry and yearling stages, HF had the greatest post-trial mortality (~55%), BW had intermediate mortality (20–25%) and the other strains (CL, FV and PN) had very low mortality. BW and CL at the yearling stage were assessed for post-trial mortality in both brood years, and post-trial mortality was consistent across years with BW incurring ~ 20% mortality and CL experiencing < 5%. This consistency is particularly striking as the experimental protocol differed between years, with 2 weeks in between ILOS and CT_max_ assessment in the 2017 brood year and 3 weeks in the 2018 brood year.

### **Correlation between CT**_**max**_  **and ILOS (experiments 2 and 3)**

**Figure 4:**
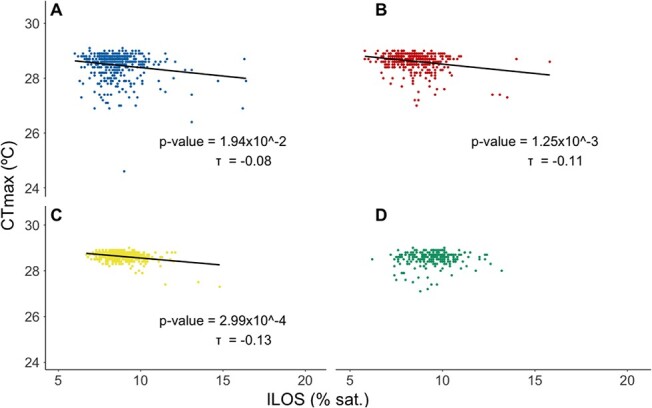
Correlation between hypoxia tolerance (ILOS) and upper thermal tolerance (CT_max_) for the fry life stage for 2018 brood (experiment 3). (**a**) BW, Blackwater strain (in blue), (**b**) CL, Carp Lake Strain (in red), (**c**) HF, Horsefly strain (in yellow); (**d**) PN, Pennask Lake strain (in green). Sample sizes differ from Tables 1 and 2 due to PIT tag loss in some individuals and are as follows: BW: *n* = 435; CL: *n* = 426; HF: *n* = 453; PN: *n* = 253. All correlations were analyzed using Kendall rank correlation (α = 0.05). Note that CT_max_ was determined 3 weeks following the determination of ILOS.

Correlations between hypoxia and upper thermal tolerance were assessed for experiments involving tagged fish. For the fry life stage (2018 brood year only) there were weak but statistically significant correlations between hypoxia and upper thermal tolerance in the BW, CL and HF strains but not PN (Fig. 4; BW: *P* = 1.94 × 10^−2^, τ = −0.08; CL: *P* = 1.25 × 10^−3^, τ = −0.11; HF: *P* = 2.99 × 10^−4^, τ = −0.13; PN: *P* = 3.62 × 10^−1^), with individuals having a high tolerance to hypoxia (low ILOS) also tending to have a high upper thermal tolerance. However, these analyses were influenced by outliers, and only the correlation in the HF strain remained significant following outlier removal (*P* = 5.84 × 10^−4^, τ = −0.10).

**Figure 5:**
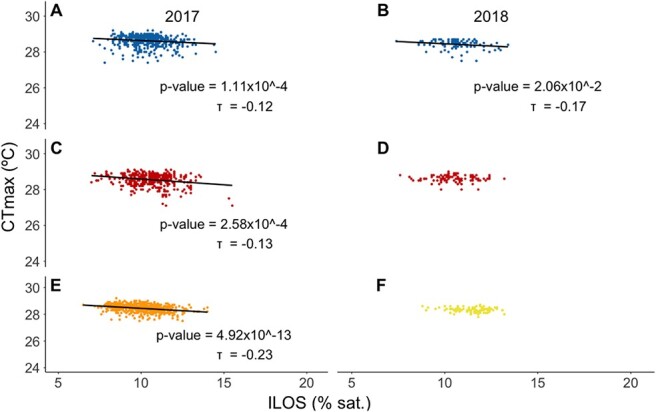
Correlation between hypoxia tolerance (ILOS) and upper thermal tolerance (CT_max_) for multiple strains across two brood years (2017 panels **a, c and e**; 2018 panels **b, d and f**) at the yearling life stage (experiment 3). (**a,b**) BW, Blackwater strain (in blue); (**c,d**) CL, Carp Lake strain (in red); (**e**) FV, Fraser Valley strain (in orange); (**f**) HF, Horsefly strain (in yellow). Sample sizes differ from Tables 1 and 2 due to PIT tag loss in some individuals and are as follows: **BW (2017):**  *n* = 499; **BW (2018):**  *n* = 100; **CL (2017):**  *n* = 400; **CL (2018):**  *n* = 100; **FV:**  *n* = 491; **HF:**  *n* = 99. All correlations were analyzed using Kendall rank correlation (α = 0.05). Note that CTmax was determined 2 weeks following the determination of ILOS in 2017 and 3 weeks following the determination of ILOS in 2018.

At the yearling stage of both the 2017 and 2018 brood years, there were weak but statistically significant correlations between hypoxia and upper thermal tolerance in BW (2017 and 2018), CL (2017 but not 2018), FV (assessed in 2017 only), with individuals having high tolerance to hypoxia also tending to have high upper thermal tolerance (2017 yearlings Fig. 5a,c,e: BW: *P* = 1.11 × 10^−4^, τ = −0.12; CL: *P* = 2.58 × 10^−4^, τ = −0.13; FV: *P* = 4.92 × 10^−13^, τ = −0.23; 2018 yearlings Fig. 5b,d,f: BW: *P* = 2.06 × 10^−2^, τ = −0.17; CL: *P* = 9.13 × 10^−1^; HF: *P* = 1.27 × 10^−1^). These correlations remained robust following outlier removal. Note that the testing protocol differed between the two years, with 2 weeks of recovery between trials for the 2017 brood year and 3 weeks of recovery between trials for the 2018 brood year.

Upon visual inspection, there was no clear relationship between post-trial mortality and either CT_max_ or ILOS in any strain when individuals that died were indicated on graphs of CT_max_ and ILOS correlations (Supplementary Fig. S3 and S4; see online supplementary material for a colour version of this figure). For the strain with the highest mortality, HF, we tested whether there was a significant difference in tolerance between fish that survived or those that ultimately died after the trial using a mixed effects model with lived/died as the main effect and trial group as a random effect. There was no significant difference in either CT_max_ or ILOS between fish that survived and fish that perished following the CT_max_ trials (Fry: *P* = 5.97 × 10^−1^ for ILOS, *P* = 2.03 × 10^−1^ for CT_max_; Yearling: *P* = 2.61 × 10^−1^ for ILOS, *P* = 6.48 × 10^−2^ for CT_max_; Supplementary Table S9).

### Effect of trial order (experiment 4)

**Figure 6:**
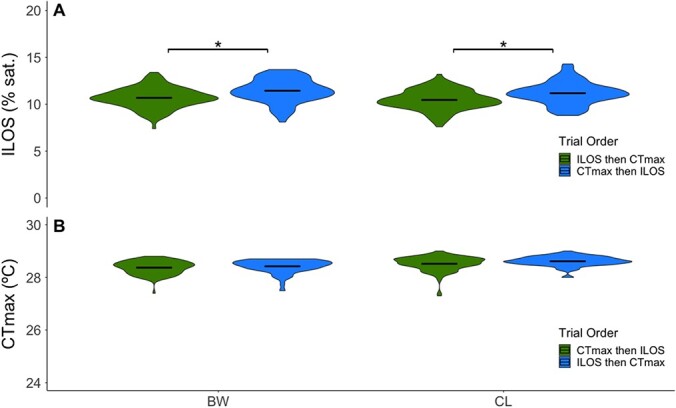
Trial order effect on hypoxia tolerance (ILOS) and upper thermal tolerance (CT_max_). (**a**) ILOS for Blackwater (BW) and Carp Lake (CL) strains, (**b**) CT_max_ for BW and CL. Black bars indicate mean of each strain. For sample sizes, see Table 3. Trial order effect on ILOS and CT_max_ was analyzed using linear mixed effects models with Tukey pairwise comparisons (α = 0.05). Significant differences between trial orders are indicated by an asterisk (*). Green for both CT_max_ and ILOS indicates fish that did not undergo a previous experiment; blue indicates fish that did undergo a previous experiment. Note that data for trials with ILOS then CT_max_ are the same as those shown in Fig. 2.

Trial order affected hypoxia tolerance in both of the strains assessed (BW and CL), with prior experience of a CT_max_ trial associated with decreased hypoxia tolerance (increased ILOS of 0.5–0.7% DO saturation; Fig. 6A, BW: *P* = 3.97 × 10^−2^, CL: *P* = 1.16 × 10^−5^). With a ramping rate of 0.1% sat min^−1^ throughout the LOE period, this absolute difference between means accounts for an ~5- to 7-minute difference in tolerance under extreme hypoxia. In contrast, having first experienced a hypoxia tolerance trial did not significantly affect upper thermal tolerance in either the CL or BW strains (Fig. 6B, BW**:**  *P* = 4.79 × 10^−1^, CL**:**  *P* = 4.31 × 10^−1^).

## Discussion

This study clearly demonstrates that different measures of tolerance to climate change stressors lead to different pictures of the relative resilience of rainbow trout strains. Although there was limited among-strain variation in CT_max_ and ILOS, the strains we studied differed greatly in post-trial mortality following tolerance assessment. These data suggest that the HF strain is quite sensitive to acute exposures to hypoxia followed by high temperature, whereas the CL and FV strains are highly tolerant. This is in contrast to the observation that all the strains have similar CT_max_ and ILOS, and the small differences we detected often resulted in a different rank-order of resilience compared to mortality. Although mean CT_max_ and ILOS were quite similar among strains, there was high inter-individual variation in CT_max_ and ILOS that could potentially allow this species to adapt to changes in temperature and aquatic oxygen that are likely to occur as a result of anthropogenic climate change. Additionally, unlike other studies in salmonids that have detected a strong correlation between CT_max_ and ILOS (e.g. Zhang *et al.*, 2018), we detect little or no relationship between these traits at the individual level, which suggests that different mechanisms underlie variation in these two traits.

Due to our robust design involving hundreds of individuals we were able to accurately assess the extent of within-strain variation in CT_max_ and ILOS. We found substantial variation in both traits, with CT_max_ varying by as much as 1.5°C and ILOS varying by as much as 5% DO saturation, excluding outliers. All strains were raised from fertilization in similar conditions, and thus these inter-individual differences in CT_max_ and ILOS may reflect genetic differences (Garland & Adolph, 1991). If the variation we observed is genetically based, then this may represent standing genetic variation upon which selection could act (Hoffmann & Sgró, 2011), which might allow rainbow trout to adapt to future warming and declines in DO. Future examination of genetic variation across individuals may be able to establish the genetic architecture of these tolerances. There were also differences in the extent of variation among strains for upper thermal tolerance, but not hypoxia tolerance, with the FV strain showing the least variation in upper thermal tolerance. The FV strain is domesticated and thus this may represent a loss of phenotypic or genetic variation during the process of domestication.

Unlike previous studies of strain-level variation in thermal tolerance in salmonids (e.g. Chen *et al.*, 2015; Scott *et al.*, 2015; Stitt *et al.*, 2014; Zhang *et al.*, 2018), we found limited and inconsistent differences in CT_max_ among strains and across life stages (Fig. 1 and 2). For example, our strains differed in mean CT_max_ by a maximum of 0.4°C, on average. This contrasts with differences of as much as 2°C among strains of rainbow trout (for fish acclimated to 15°C) reported in an analysis of data across multiple previous studies (Chen *et al.*, 2015). One possible explanation for this difference is that these previous studies used a range of thermal ramping rates, were performed in different locations, at different times of the year, and on fish ranging in size from 2 to 140 g. Alternatively, the limited variation in CT_max_ that we observed among our strains may be explained by the fact that, with the exception of FV, all of the strains we used are from a rainbow trout lineage from the interior of British Columbia (Holmes, 2009; McCusker, Parkinson, & Taylor, 2000; Pollard & Yesaki, 2004; Tamkee, Parkinson, & Taylor, 2010; Taylor, Tamkee, Keeley, & Parkinson, 2011). However, this cannot explain the observation of limited differences in CT_max_ between the wild British Columbia strains and the FV domesticated strain, which is thought to be of California origin and might be expected to have higher thermal tolerance. Indeed, the low post-trial mortality observed with this strain is consistent with a greater resilience to climate change in this more southern lineage. However, comparisons with this strain must be viewed with caution because it was tested at a different time of year from the other strains in our experiments. Another important consideration is that the FV strain has been used in the BC stocking programme since 1960s and was initially domesticated in the 1940s (Northrup, 2017). The extent of introgression of alleles from other strains and the role of long-term selection in a constant-temperature hatchery environment in determining the CT_max_ of this strain remain unknown. The limited differences among strains in CT_max_ that we observe, taken together with the relatively limited differences in this trait across studies (reviewed in Chen *et al.*, 2015), suggest that assessment of CT_max_ alone is not likely to be the most useful tool for detecting strains that are particularly resilient to climate change stressors.

As was the case for CT_max_, we also detected relatively small differences in ILOS among our strains of rainbow trout, with LOE occurring at 10–11.4% DO saturation in yearlings and 8.6–8.9% saturation in fry (Supplementary Tables S5 and S8). Fewer studies have examined variation in hypoxia tolerance among strains in rainbow trout, but Scott *et al.* (2015) assessed time to LOE in rainbow trout fry and yearling at 10% DO saturation, which is conceptually similar to our analysis of ILOS. They found differences between the strains of ~10 min in time to LOE (on average). Taking into account the oxygen ramping rate in our trials, the difference in ILOS among our strains was similar at ~10-min difference in time to LOE for yearlings and 3-min difference for fry. This relatively small difference, compared to within-strain variation, suggests rainbow trout strains may be similar in their acute hypoxia tolerance.

In contrast to the limited differentiation among strains in upper thermal and hypoxia tolerance, we found substantial and consistent differences in post-trial mortality among our strains. The FV, PN and CL strains exhibited little post-trial mortality (1–4% depending on the strains), the BW strain exhibited intermediate mortality (~20%), whereas the HF strain exhibited extreme mortality (~55%). This high level of mortality is unusual following a CT_max_ trial, as post-trial mortality is generally thought to be low (1–5%) in fish (Anttila *et al.*, 2013; Joyce & Perry, 2020; Morgan *et al.*, 2018; Scott *et al.*, 2015; Zhang *et al.*, 2018). The main difference between our study and most others is that our fish were exposed to a hypoxia tolerance trial 2–3 weeks previous to the measurement of thermal tolerance, which might contribute to additional mortality. However, this cannot explain the substantial differences in mortality among strains. In general, the HF strain was a poor performer in most of the metrics we assessed, with relatively low CT_max_ and high ILOS (Fig. 2 and Supplementary Table S8), although these differences in acute tolerance were much less clear than the differences in post-trial mortality.

It is possible that the relatively poor performance of the HF strain at high temperature or low oxygen as well as its high post-trial mortality is a consequence of local adaptation to its native environment. HF spawn in the lower Horsefly River, but reside in Quesnel Lake, a very deep (~157 m average depth) fjord lake that rarely experiences temperatures > 18°C (Petticrew *et al.*, 2015; Stiff, Hyatt, Cone, Patterson, & Benner, 2018), while the other strains originate from, or are kept as broodstock in, lakes that regularly experience temperatures > 22°C with temperatures exceeding 25°C in some habitats (data not shown). Furthermore, deep water in Quesnel Lake is generally near full DO saturation (James, Laval, Carmack, & Pieters, 2004); therefore, HF trout rarely experience extreme thermal or hypoxia exposure and may be less able to cope with these stressors, potentially explaining their high mortality following stressor exposure. Overall, these data clearly demonstrate that measurements of ILOS or CT_max_ do not fully capture the variation in sensitivity among strains. In addition, these measures reflect variation in sensitivity to acute, extreme stressful events, and it is possible that chronic exposure to less extreme conditions may be ecologically more relevant (McKenzie *et al.*, 2020). These data add to the growing consensus that more nuanced approaches are required to assess the thermal and oxic niches of fish strains in the context of climate change (Åsheim *et al.*, 2020; McKenzie *et al.*, 2016).

Our data suggest that there is little to no relationship between whole-animal upper thermal tolerance (assessed as CT_max_) and hypoxia tolerance (assessed as ILOS) when compared across individuals within a strain. This is in contrast to previous studies, which have indicated that there is a relationship between these traits in a variety of species of salmonids (Anttila *et al.*, 2013; Zhang *et al.*, 2018), which has been used to suggest that there may be a mechanistic link between variation in these two traits; however, these previous studies compared rank order across families or strain rather than across individuals. Similar to our findings, Joyce and Perry (2020) found that CT_max_ was not correlated with hypoxia tolerance across individuals in zebrafish, further supporting the lack of direct mechanistic linkage between these traits. Indeed, evidence is accumulating that the mechanisms underlying acute upper thermal and hypoxia tolerance are likely very different (Jutfelt *et al.*, 2019; Mandic, Best, & Perry, 2020). The knockout of HIF-1α in zebrafish, a protein implicated in cellular metabolism (Semenza, 2012), clearly shows this difference as it results in declines in hypoxia tolerance but not thermal tolerance (Mandic *et al.*, 2020). Thus, oxygen transport processes or metabolic regulation is likely critical in determining hypoxia tolerance. In contrast it is likely that failure of neurological mechanisms, not oxygen transport (Wang *et al.*, 2014), may be responsible for setting acute thermal tolerance (Jutfelt *et al.*, 2019).

Natural environments are complex and involve changes in multiple interacting stressors, and thus environmentally relevant assessments of climate change resilience should involve determining tolerance to multiple stressors. Indeed, it is becoming increasingly common to measure multiple traits in individual fish to obtain a multifaceted view of the responses of organisms to the environment (Åsheim *et al.*, 2020; Gunderson, Armstrong, & Stillman, 2016; Joyce & Perry, 2020; Nudds, Ozolina, Fenkes, Wearing, & Shiels, 2020). However, measuring multiple traits in single individuals results in logistical challenges as prior exposure to a stressful environment has the potential to alter subsequent tolerance, either reducing tolerance due to accumulation of cellular or organismal damage or improving tolerance through phenomena such as heat-hardening and cross-tolerance (McArley, Hickey, & Herbert, 2020; McBryan, Anttila, Healy, & Schulte, 2013; Morgan *et al.*, 2018; Todgham *et al.*, 2005). For example, Todgham *et al.* (2005) found that prior exposure to heat shock improved hypoxia tolerance in fish. However, McArley *et al.* (2020) found opposite results, with a longer heat-shock impairing subsequent hypoxia tolerance. Similarly, it has been suggested that the interaction between temperature and hypoxia can impair tolerance (McBryan *et al.*, 2013). Few, if any, studies have examined the reciprocal effects of previous temperature or hypoxia exposure on these respective tolerances. Here, we show that there is no effect of prior exposure to hypoxia on upper thermal tolerance but that there are significant decreases in hypoxia tolerance if individuals had previously experienced a thermal tolerance trial (Fig. 4). This suggests that exposure to high temperatures during a CT_max_ trial is more physiologically stressful than exposure to hypoxia during an ILOS trial. This conclusion is also supported by the fact that little or no mortality was experienced in the 2–3 weeks following the ILOS trial, but (at least in some strains) there was considerable mortality following the CT_max_ trial. These results emphasize the importance of careful experimental design in studies assessing multiple tolerance metrics in individual fish and provide important lessons for the design of studies in conservation physiology going forward. In addition, our experiments only assessed the effects of acute exposure to extremes of temperature and hypoxia, and intraspecific variations in the response to chronic stressors or the ability to recover from stressful events are likely to also be important in an organism’s natural environment.

## Conclusion

Incorporating physiological information into fisheries management strategies is increasingly important in the context of the effects of anthropogenic climate change (Ficke *et al.*, 2007; Madliger *et al.*, 2016; McKenzie *et al.*, 2016). Moreover, it is becoming increasingly common for fishery and conservation managers to use physiological measurements to examine individual, strain and species-specific responses (Harrod, 2016; Madliger *et al.*, 2016). Here we show that different metrics of tolerance (e.g. hypoxia or upper thermal tolerance vs post-trial mortality) provide different information. This observation has important direct implications for the management of rainbow trout, an economically important freshwater fish, but are also generalizable across fish species and this lesson is likely to be transferrable to the assessment of climate change resilience in a wide variety of organisms. Another important lesson from the data presented here is the power that can be obtained using a common garden approach and very large sample sizes. Taken together, these results are an important additional input into the multi-faceted decision-making process required to plan stocking programmes in the face of climate change and to conserve strains of this important recreational fish species (Madliger *et al.*, 2016; McKenzie *et al.*, 2016; Reid *et al.*, 2019).

## Author Contributions

P.M.S., S.L.N. and N.S. designed the study; N.S., S.L.N., M.L.E. and T.S.B. conducted the study; P.M.S., N.S., M.L.E. and T.S.B. analyzed the data and all authors contributed to the writing of the manuscript.

## Funding

This work was supported by a Genome Canada and Genome British Columbia Large Scale Applied Research Project (grant number 24RTE): Sustaining Freshwater Recreational Fisheries in a Changing Environment.

## Supplementary Material

supp_coab095
