# Supplementary material for: Acute measures of upper thermal and hypoxia tolerance are not reliable predictors of mortality following environmental challenges in rainbow trout (Oncorhynchus mykiss)
Source: Conserv Physiol. 2021 Dec 23;9(1):coab095. doi: 10.1093/conphys/coab095 (PMC8710852; doi:10.1093/conphys/coab095)
Supplement: supp_coab095 [file supp_coab095.zip › Strowbridge et al. Nov 16 2021 supplemental figures and tables.docx]

Supplemental materials


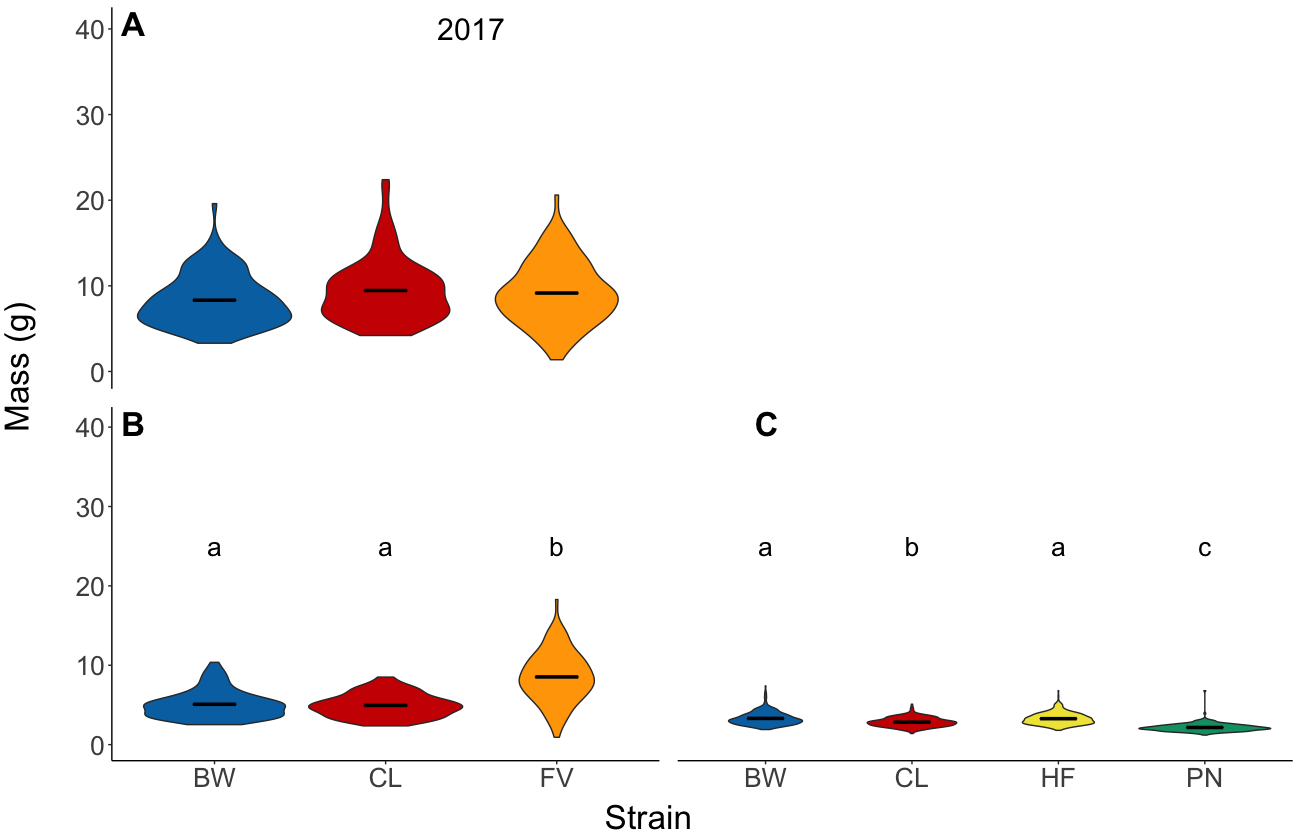


**Supplemental Figure 1:** Mass of fry from 2017 and 2018 brood years (Experiments 1, 2 and 3). Panels **A, B** mass of fish from trials where ILOS and CT_max­_ were conducted on different fish, Panel **C** mass of fish exposed to both ILOS and CT_max_ trials. BW= Blackwater strain (in blue), CL= Carp Lake strain (in red), FV = Fraser Valley strain (in orange), HF = Horsefly strain (in yellow), PN = Pennask Lake strain (in green). Black bars indicate mean of each strain. Significant differences between strains are indicated by dissimilar letters. All data were analyzed one-way ANOVA with Tukey-HSD pairwise comparisons (α = 0.05). See Tables 1 and 2 for sample sizes.


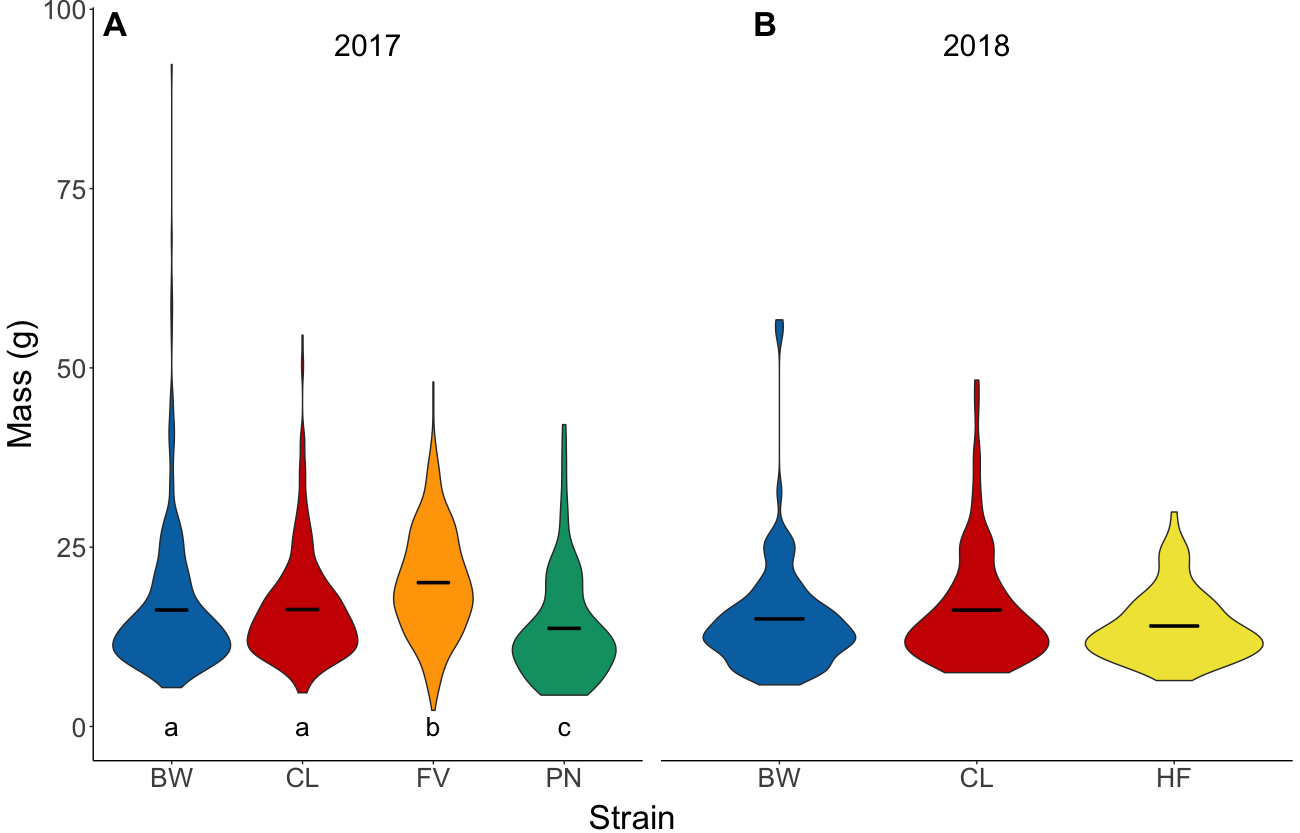


**Supplemental Figure 2:** Mass for individual strains at the yearling life-stage (Experiments 2 and 3). Panel **A** mass of fish exposed to both ILOS and CT_max_ trials in 2017 brood year; Panel **B** mass of fish exposed to both ILOS and CT_max_ trials in 2018 brood year. BW= Blackwater strain (in blue). CL= Carp Lake strain (in red), FV = Fraser Valley strain (in orange), HF = Horsefly strain (in yellow), PN = Pennask Lake strain (in green). Black bars indicate mean of each strain. See Table 2 for sample sizes. All data were analyzed one-way ANOVA with Tukey-HSD pairwise comparisons (α = 0.05). See Tables 1 and 2 for sample sizes.


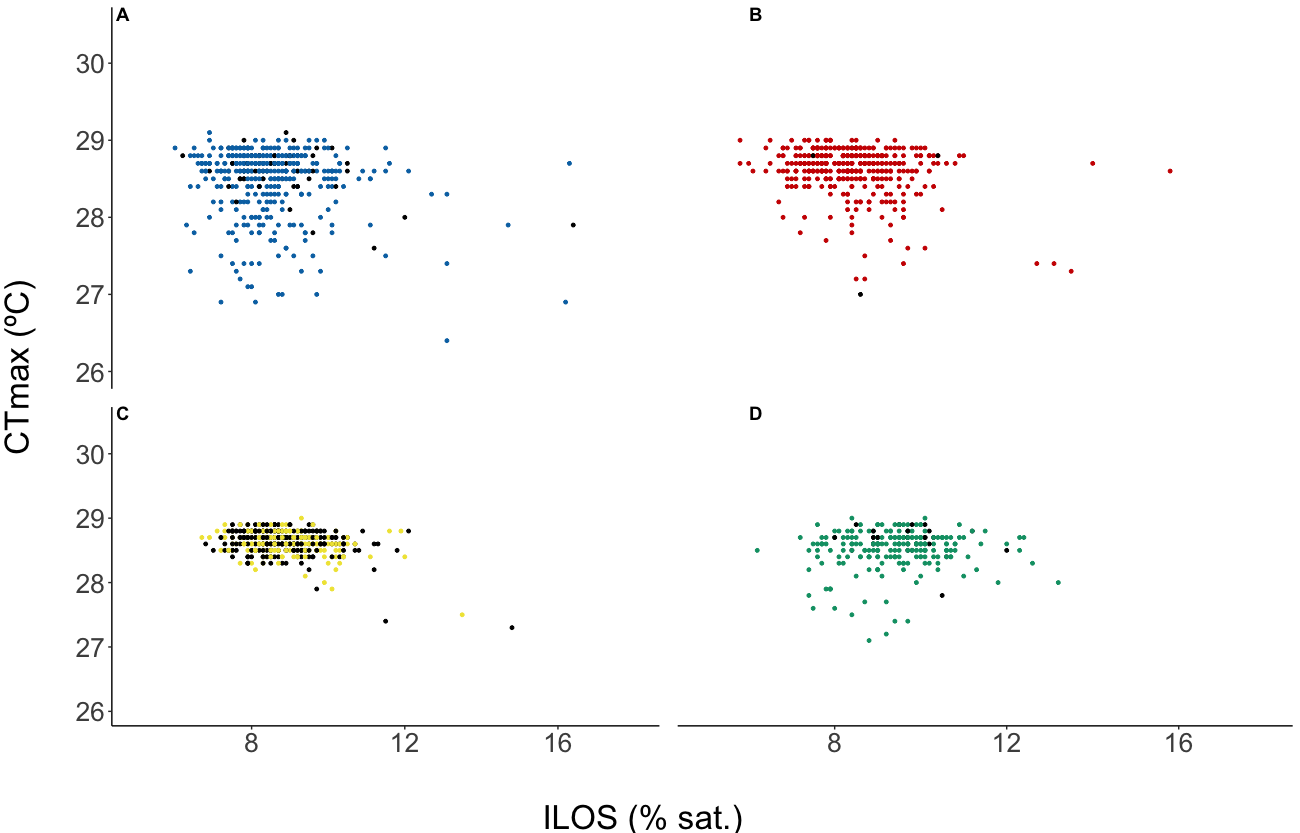


**Supplemental Figure 3:** Mortality mapped onto critical thermal maximum (CT_max_) and incipient lethal oxygen saturation (ILOS) for the fry life-stage for 2018 brood. Individuals that died following CT_max_ are coloured black. **A** BW = Blackwater strain (in blue), **B** CL = Carp Lake Strain (in red), **C** HF = Horsefly strain (in yellow), **D** PN = Pennask Lake strain (in green). Sample sizes differ from Tables 1 and 2 due to PIT tag loss in some individuals and are as follows: **BW:** n = 435, **CL:** n = 426, **HF:** n = 453, **PN:** n = 253.


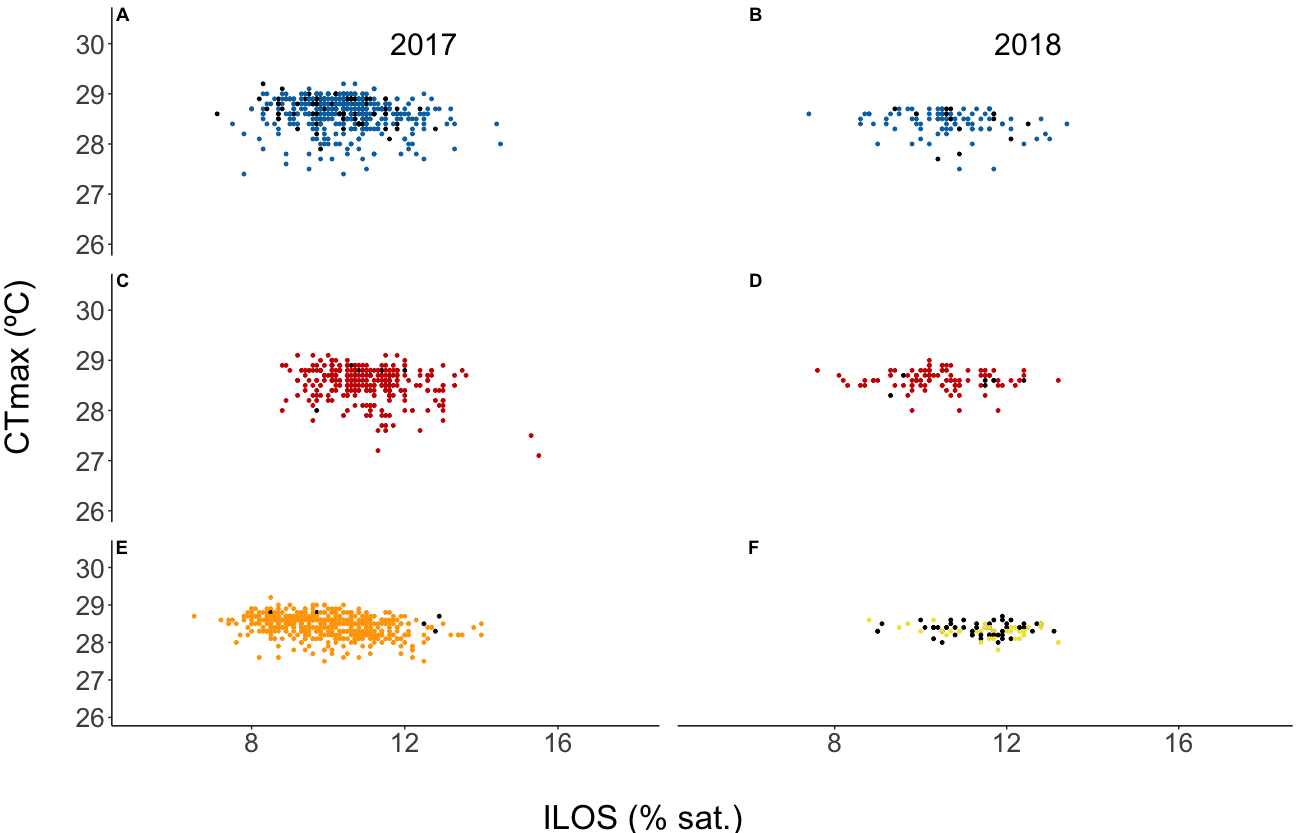


**Supplemental Figure 4:** Mortality mapped onto critical thermal maximum (CT_max_) and incipient lethal oxygen saturation (ILOS) for the yearling life-stage across two brood years (2017, 2018). Individuals that died following CT_max_ are coloured black. **A, B** BW = Blackwater strain (in blue), **C,D** CL = Carp Lake strain (in red), **E** FV = Fraser Valley strain (in orange), **F** HF = Horsefly strain (in yellow). Sample sizes differ from Tables 1 and 2 due to PIT tag loss in some individuals and are as follows: **BW (2017):** n = 499, **BW (2018):** n = 100, **CL (2017):** n = 400, **CL (2018):** n = 100, **FV:** n = 491, **HF:** n = 99.

**Supplemental Table 1:** Mean ± standard deviation of mass for strains in the 2017 brood year

| **Strain** | **Fry**  **(grams)** | | **Yearling**  **(grams)** |
| --- | --- | --- | --- |
|  | **Hypoxia** | **Thermal** |  |
| **Blackwater River (BW)** | 8.32 ± 2.99 | 5.08 ± 1.74^a^ | 16.20 ± 9.34^a^ |
| **Carp Lake (CL)** | 9.45 ± 3.67 | 4.94 ± 1.46^a^ | 16.30 ± 7.32^a^ |
| **Fraser Valley (FV)** | 9.16 ± 3.65 | 8.53 ± 3.17^b^ | 20.10 ± 7.50^b^ |
| **Pennask Lake (PN)** | NA | NA | 13.70 ± 7.52^c^ |

Significant differences between groups within a strain are indicated by dissimilar letters

**Supplemental Table 2:** Mean ± standard deviation of mass for strains in the 2018 brood year

| **Strain** | **Fry**  **(grams)** | **Yearling**  **(grams)** |
| --- | --- | --- |
| **Blackwater River (BW)** | 3.31 ± 0.82^a^ | 15.00 ± 7.88 |
| **Carp Lake (CL)** | 2.83 ± 0.59^b^ | 16.20 ± 7.90 |
| **Horsefly River (HF)** | 3.28 ± 0.72^a^ | 14.00 ± 4.83 |
| **Pennask Lake (PN)** | 2.14 ± 0.49^c^ | NA |

Significant differences between groups within a strain are indicated by dissimilar letters

**Supplemental Table 3:** Statistical results from the correlation of CT_max_ or ILOS vs. mass for all strains at the fry stage

| **Strain** | | **P-value** | | | **Correlation coefficient** | | |
| --- | --- | --- | --- | --- | --- | --- | --- |
|  |  | **ILOS (% sat.)** | **CT_max_ (°C)** | | **ILOS (% sat.)** | | **CT_max_ (°C)** |
| **Blackwater River:** | **2017** | 0.938 | 0.976 | -0.005 | | 0.002 | |
|  | **2018** | 0.112 | 0.316 | -0.050 | | -0.034 | |
| **Carp Lake:** | **2017**  **2018** | 0.594 | 0.190 | 0.037 | | 0.093 | |
|  |  | 0.876 | 0.102 | -0.005 | | 0.058 | |
| **Fraser Valley:** | **2017** | 0.005 | 0.002* | 0.281 | | -0.310 | |
|  | **2018** | - | - | - | | - | |
| **Horsefly River:** | **2017** | - | - | - | | - | |
|  | **2018** | 0.008 | 0.003* | -0.082 | | 0.104 | |
| **Pennask Lake:** | **2017** | - | - | - | | - | |
|  | **2018** | 0.187 | 0.467 | 0.041 | | 0.033 | |

*indicates significant correlation after correcting for multiple comparisons

**Supplemental Table 4:** Statistical results from the correlation of CT_max_ or ILOS vs. mass for all strains at the yearling stage

| **Strain** | | **P-value** | | | **Correlation coefficient** | | |
| --- | --- | --- | --- | --- | --- | --- | --- |
|  |  | **ILOS (% sat.)** | **CT_max_ (°C)** | | **ILOS (% sat.)** | | **CT_max_ (°C)** |
| **Blackwater River:** | **2017** | 0.027 | 0.515 | -0.067 | | -0.021 | |
|  | **2018** | 0.128 | 0.088 | 0.104 | | -0.123 | |
| **Carp Lake:** | **2017**  **2018** | 0.002* | 0.056 | 0.106 | | -0.0674 | |
|  |  | 0.006 | 0.531 | 0.188 | | -0.046 | |
| **Fraser Valley:** | **2017** | 5.30x10^-4^* | 1.01x10^-7^* | 0.154 | | -0.236 | |
|  | **2018** | - | - | - | | - | |
| **Horsefly River:** | **2017** | - | - | - | | - | |
|  | **2018** | 0.788 | 0.327 | -0.019 | | 0.072 | |
| **Pennask Lake:** | **2017** | 0.015 | 0.005 | -0.168 | | 0.203 | |
|  | **2018** | - | - | - | | - | |

*indicates significant correlation after correcting for multiple comparisons

**Supplemental Table 5:** Mean ± standard deviation of ILOS and CT_max_ for strains in the 2017 brood year

| Strain | Fry | | Yearling | |
| --- | --- | --- | --- | --- |
|  | **ILOS (% sat.)** | **CT_max_ (°C)** | **ILOS (% sat.)** | **CT_max_ (°C)** |
| Blackwater River (BW) | 12.1 ± 1.2 | 28.2 ± 0.5 | 10.4 ± 1.1^a^ | 28.6 ± 0.3^a^ |
| Carp Lake (CL) | 11.3 ± 1.1 | 28.4 ± 0.4 | 11.0 ± 1.0^b^ | 28.5 ± 0.3^ac^ |
| Fraser Valley (FV) | 9.54 ± 1.2 | 28.6 ± 0.2 | 10.0 ± 1.3^c^ | 28.4 ± 0.3^c^ |
| Pennask Lake (PN) | NA | NA | 10.3 ± 1.3 | 28.3 ± 0.6 |

Significant differences between groups within a strain are indicated by dissimilar letters

Note: Statistical comparisons were not conducted for the fry stage. Pennask Lake strain was not include in statistical comparisons for the yearling stage. See Table 1 for sample sizes.

**Supplemental Table 6:** Individual trial incipient lethal oxygen saturation (% sat.) comparisons for strains at the 2017 brood year yearling stage

| Trial # | Blackwater River  (mean ± sd) | Carp Lake  (mean ± sd) | Fraser Valley  (mean ± sd) |
| --- | --- | --- | --- |
| 1 | 10.6 ± 1.4 % sat. | 11.2 ± 0.8 % sat.^a^ | 9.8 ± 1.4 % sat.^a^ |
| 2 | 10.3 ± 1.1 % sat. | 10.7 ± 1.0 % sat.^b^ | 9.8 ± 1.1 % sat.^ab^ |
| 3 | 10.3 ± 1.0 % sat. | 11.2 ± 1.2 % sat.^a^ | 10.0 ± 1.2 % sat.^ab^ |
| 4 | 10.2 ± 1.0 % sat. | 9.5 ± 1.1 % sat.^c^ | 10.3 ± 1.2 % sat.^b^ |
| 5 | 10.5 ± 1.0 % sat. | NA | 10.1 ± 1.3 % sat.^ab^ |

Significant differences between groups within a strain are indicated by dissimilar letters

Note: n = 99-101 fish per trial

**Supplemental Table 7:** Individual trial critical thermal maximum (°C) comparisons for strains at the 2017 brood year yearling stage

| Trial # | Blackwater River  (mean ± sd) | Carp Lake  (mean ± sd) | Fraser Valley  (mean ± sd) |
| --- | --- | --- | --- |
| 1 | 28.6 ± 0.3 °C | 28.5 ± 0.3 °C^a^ | 28.5 ± 0.3 °C^a^ |
| 2 | 28.6 ± 0.3 °C | 28.4 ± 0.3 °C^a^ | 28.5 ± 0.3 °C^a^ |
| 3 | 28.6 ± 0.3 °C | 28.6 ± 0.3 °C^b^ | 28.4 ± 0.3 °C^ab^ |
| 4 | 28.6 ± 0.3 °C | 28.6 ± 0.3 °C ^b^ | 28.4 ± 0.3 °C^ab^ |
| 5 | 28.7 ± 0.3 °C | NA | 28.3 ± 0.3 °C^b^ |

Significant differences between groups within a strain are indicated by dissimilar letters

Note: n = 99-100 fish per trial

**Supplemental Table 8:** Mean ± standard deviation of ILOS and CT_max_ for strains in the 2018 brood year

| Strain | Fry | | Yearling | |
| --- | --- | --- | --- | --- |
|  | **ILOS (% sat.)** | **CT_max_ (°C)** | **ILOS (% sat.)** | **CT_max_ (°C)** |
| Blackwater River (BW) | 8.6 ± 1.3^a^ | 28.5 ± 0.5^a^ | 10.7 ± 1.1^a^ | 28.4 ± 0.3^a^ |
| Carp Lake (CL) | 8.5 ± 1.2^a^ | 28.6 ± 0.3^b^ | 10.5 ± 1.1^a^ | 28.6 ± 0.2^b^ |
| Horsefly River (HF) | 8.9 ± 1.0^b^ | 28.6 ± 0.2^c^ | 11.4 ± 1.0^b^ | 28.3 ± 0.2^c^ |
| Pennask Lake (PN) | 9.5 ± 1.2 | 28.5 ± 0.3 | NA | NA |

Significant differences between groups within a strain are indicated by dissimilar letters

Note: Pennask Lake strain was not include in statistical comparisons for the fry stage. See Table 2 for sample sizes.

**Supplemental Table 9:** Mean ± standard deviation of ILOS and CT_max_ for fish that died (mortality) and fish that did not die (non-mortality) following CT_max­ ­_trials for the Horsefly strain

| Strain | Fry | | Yearling | |
| --- | --- | --- | --- | --- |
|  | **ILOS (% sat.)** | **CT_max_ (°C)** | **ILOS (% sat.)** | **CT_max_ (°C)** |
| Mortality | 8.88 ± 1.0 | 28.6 ± 0.2 | 11.3 ± 0.9 | 28.4 ± 0.2 |
| Non-mortality | 8.85 ± 1.0 | 28.6 ± 0.2 | 11.5 ± 1.0 | 28.3 ± 0.2 |

Note: **Fry:** Mortality n = 276, Non-mortality n = 177. **Yearling:** Mortality n = 53, Non-mortality n = 99.
